# Association between Cognitive Impairment and Hippocampal Subfield Volumes in Multiple System Atrophy

**DOI:** 10.1155/2023/8888255

**Published:** 2023-03-06

**Authors:** Atsuhiko Sugiyama, Hajime Yokota, Shigeki Hirano, Jiaqi Wang, Shoichi Ito, Satoshi Kuwabara

**Affiliations:** ^1^Department of Neurology, Graduate School of Medicine, Chiba University, Chiba, Japan; ^2^Department of Diagnostic Radiology and Radiation Oncology, Graduate School of Medicine, Chiba University, Chiba, Japan; ^3^Department of Medical Education, Graduate School of Medicine, Chiba University, Chiba, Japan

## Abstract

This study aimed to explore morphological changes of hippocampal subfields in patients with multiple system atrophy (MSA) with and without cognitive impairment using FreeSurfer-automated segmentation of hippocampal subfield techniques and their relationship with cognitive function. We enrolled 75 patients with MSA classified as cognitively impaired MSA (MSA-CI, *n* = 40) and cognitively preserved MSA (MSA-CP, *n* = 35), as well as 68 healthy controls. All participants underwent three-dimensional volume T1-weighted magnetic resonance imaging. The hippocampal subfield volume was measured using FreeSurfer version 7.2 and compared among groups. Regression analyses were performed between the hippocampal subfield volumes and cognitive variables. Compared with healthy controls, the volume of the right cornu ammonis (CA) 2/3 was significantly lower in the MSA-CI group (*P*=0.029) and that of the left fimbria was significantly higher in the MSA-CP group (*P*=0.046). Results of linear regression analysis showed that the right CA2/3 volume was significantly correlated with the Frontal Assessment Battery score in patients with MSA (adjusted *R*^2^ = 0.282, *β* = 0.227, and *P*=0.041). The hippocampal subfield volume decreased in patients with MSA-CI, even at the early disease stages. Specific structural changes in the hippocampus might be associated with cognitive deficits in MSA.

## 1. Introduction

Multiple system atrophy (MSA) is a progressive neurodegenerative disorder characterized by various combinations of autonomic failure, cerebellar ataxia, and parkinsonism. Pathologically, glial cytoplasmic inclusions (GCIs) and neuronal cytoplasmic/nuclear inclusions (NCIs/NNIs) are found throughout the brain, especially in striatonigral and olivopontocerebellar systems [1, 2]. Cognitive impairment is traditionally believed to rarely develop in MSA [3]; however, accumulating evidence has indicated that cognitive dysfunction is an integral part of MSA [4]. The frontal executive function is most commonly affected, whereas attention, memory, language, and visuospatial functions are sometimes impaired [4].

Recent studies have indicated that the NCI burden rather than GCI in limbic regions is one of the pathological substrates of cognitive impairment in MSA [5–10]. It is currently debatable whether the NCI burden in limbic regions is specifically associated with the memory domain in cognitive function [5–9]. A longitudinal voxel-based morphometry study reported an atrophic progression in multiple brain regions, including the hippocampus [11]. However, the hippocampus consists of distinctive subfields, including the cornu ammonis (CA) 1–4, dentate gyrus, subiculum, and fimbria, which are histologically heterogeneous, with varied vulnerabilities to aging, and are functionally specialized [12]. Although a study using automated segmentation of hippocampal subfield techniques revealed that hippocampal subfield impairment is common in MSA patients [13], this study included only 30 MSA patients with mild cognitive impairment and a mean disease duration of 4 years, and it did not include a detailed evaluation of memory in the assessment of cognitive function. Therefore, there is insufficient evidence for structural changes in the hippocampal subfield and its relationship to cognitive function, particularly memory, in a larger number of MSA patients, including those in the early disease stage or who do not have mild cognitive impairment.

In this study, morphological changes of hippocampal subfields in patients with MSA in the early disease stage with and without cognitive impairment and their relationship with cognitive function including memory were investigated using FreeSurfer-automated segmentation techniques.

## 2. Materials and Methods

### 2.1. Patients

This retrospective study was approved by the Institutional Review Board of the Chiba University Graduate School of Medicine, and the need for informed consent for patients with MSA was waived. All healthy controls (HCs) provided written informed consent. From our database, we identified 83 consecutive patients with MSA who were admitted to Chiba University Hospital between September 2017 and August 2021. The inclusion criterion was patients who met the criteria for clinically possible or probable MSA, as described in the second consensus statement by Gilman et al. [14]. Diagnosis of possible or probable MSA was confirmed by a movement disorder specialist at our center. The exclusion criteria were as follows: (1) current or previous history of another neuropsychiatric disorder and (2) abnormal MRI findings due to another etiology. A total of eight patients were then excluded (three with depression, one with hemorrhage in the putamen, two with brain infarction, one with polymicrogyria on brain MRI, and one with infantile paralysis). Finally, 75 MSA patients (probable 54, possible 21) were included in the present study. Based on the predominant clinical symptomatology at the time of MRI, 44 patients were classified as having cerebellar ataxia-predominant MSA (MSA-C) and 31 as having parkinsonism-predominant MSA (MSA-P). In this study, 40 patients with MSA with Frontal Assessment Battery (FAB) scores of ≤14 were classified as cognitively impaired MSA (MSA-CI) and 35 patients with scores of >14 were classified as cognitively preserved MSA (MSA-CP) [15]. Twenty-six patients with MSA with one or more Wechsler Memory Scale-Revised (WMS-R) indices of ≤77.5 (−1.5 standard deviations) were classified as memory-impaired MSA (MSA-MI), and 49 patients with WMS-R indices of >77.5 were classified as memory-preserved MSA (MSA-MP) [16]. Sixty-nine healthy participants who volunteered in response to a local advertisement were recruited as HCs. These healthy participants had no history of neurological or psychiatric illnesses, had normal neurological examination results, and had a Mini-Mental State Examination score of ≥26.

The medical records of patients with MSA were reviewed to determine their ages at the time of an MRI scan, disease duration (time from onset to an MRI scan), the Unified Multiple System Atrophy Rating Scale (UMSARS) scores, the Zung Self-Rating Depression Scale (SDS) scores, Frontal Assessment Battery (FAB) scores, Addenbrooke's Cognitive Examination III (ACE-III) [17], and WMS-R indices.

### 2.2. MRI Acquisition

All MRI examinations were performed using a single 3-T MRI system (GE DISCOVERY MR750, GE Healthcare). Imaging parameters for T1-weighted images were 3D-IR-SPGR; sagittal plane; TR, 8 ms; TE, 3 ms; TI, 420 ms; flip angle, 15°; FOV, 256 mm; matrix, 256 × 256; and voxel size, 1 × 1 × 1 mm.

### 2.3. Imaging Data Preprocessing

The structural T1-weighted image data were processed using FreeSurfer 7.2 (http://surfer.nmr.mgh.harvard.edu/). First, several preprocessing steps were performed to reconstruct the cortical and subcortical regions, including nonbrain tissue removal, automated Talairach transformation, intensity normalization, white and gray matter volume segmentation in subcortical regions, and tessellation of the gray matter/white matter and gray matter/cerebrospinal fluid boundaries. After the abovementioned processing, the results of cortical and subcortical structural segmentation were visually inspected and corrected as necessary for each participant. Subsequently, hippocampal subsegmentation was performed using an automatic segmentation function of FreeSurfer version 7.2. The detailed process of hippocampal subfield volumetric analysis has been previously described [18]. One HC was excluded during the visual inspection of processed images due to gross errors in hippocampal subfield segmentation.

**Figure 1:**
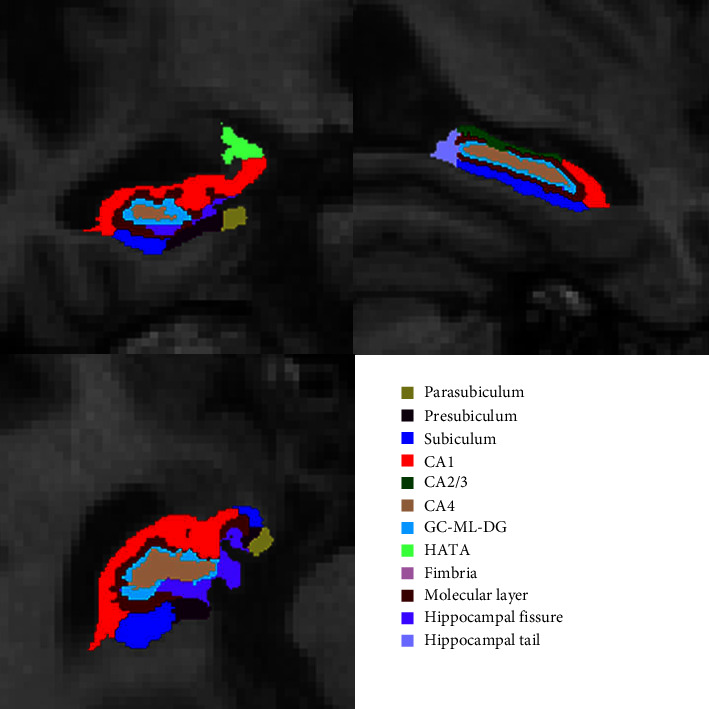
Sample of left hippocampal subfield automated segmentation. CA, cornu ammonis; GC-ML-DG, granular cell-molecular layer-dentate gyrus; HATA, hippocampus-amygdala transition area.

The hippocampus was divided into 12 subregions: hippocampal tail, subiculum, CA1, hippocampal fissure, presubiculum, parasubiculum, molecular layer, granular cell-molecular layer-dentate gyrus (GC-ML-DG), CA2/3, CA4, fimbria, and hippocampus-amygdala transition area (HATA) (Figure 1). The overall bilateral hippocampus volume, each subfields' volume, and estimated total intracranial volume (eTIV) were obtained.

### 2.4. Statistical Analysis

All statistical analyses, except for the Steel-Dwass test, were performed using SPSS software ver. 25.0 (SPSS Japan, Tokyo, Japan). The Steel–Dwass test was performed using JMP pro 14.2.0 (SAS Institute). Demographic variables from MSA-CI, MSA-CP, and HC patients were compared using the *χ*2 test for sex and disease subtypes, and the Kruskal–Wallis one-way analysis of variance with the post hoc Steel–Dwass test was adjusted for multiple comparisons for age at MRI and education. One-way analysis of variance was used to compare eTIV among groups. Disease duration, UMSARS part 2 scores, FAB, and the visual memory index of WMS-R were compared between MSA-CI and MSA-CP using the Mann–Whitney *U* test. WMS-R indices, except for visual memory, and SDS were compared between MSA-CI and MSA-CP using Student's *t*-test.

Analysis of covariance with controlled age, sex, education, and eTIV was used for group comparisons in hippocampal subfield volumes, with the post hoc Bonferroni correction (multiple testing correction for comparisons among the three groups). Hippocampal subfield volumes that were found to be statistically significant in group comparisons were included in linear regression analyses to evaluate the correlation between cognitive variables and subfield volumes in patients with MSA. Cognitive variables (FAB, total, and subscale ACE-III scores and five memory domains extracted from WMS-R results) were used as independent variables with adjustment for sex, age, disease duration, UMSARS part 2 scores, SDS, and educational background. The significance level was set at *P* < 0.05.

## 3. Results

### 3.1. Participants' Clinical Characteristics

The clinical characteristics of study participants are summarized in Table 1. Compared to MSA-CP and MSA-CI groups, the HC group had a higher proportion of women. Age at an MRI scan was significantly higher in the MSA-CI group than that in the MSA-CP and HC groups. The HC group's educational background was significantly longer than that of the MSA-CI group. Neuropsychological test results demonstrated significantly lower FAB, total, and subscale ACE-III scores and WMS-R indices in the MSA-CP and MSA-CI groups than those in the MSA-CI group.

### 3.2. Comparison of Hippocampal Subfield Volumes among the HC, MSA-CP, and MSA-CI Groups

**Figure 2:**
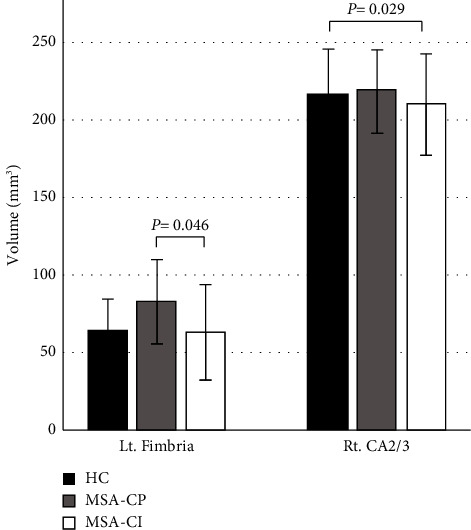
Comparisons of hippocampal subfield volume among the healthy control (HC), cognitively preserved multiple system atrophy (MSA-CP), and cognitively impaired multiple system atrophy (MSA-CI) groups. Error bars represent standard deviation. CA, cornu ammonis.

Compared with the HC group, the right CA2/3 volume was significantly lower in the MSA-CI group (215.6 ± 30.1 vs. 209.8 ± 32.5, *P*=0.029) whereas that of the left fimbria was significantly higher in the MSA-CP group (64.6 ± 20.1 vs. 83.0 ± 27.1, *P*=0.046) (Table 2 and Figure 2). No significant difference was observed in the hippocampal subfield volume between the MSA-CI and MSA-CP groups.

### 3.3. Comparison of Hippocampal Subfield Volumes among the HC, MSA-MP, and MSA-MI Groups

No significant difference was observed in the hippocampal subfield volume among the HC, MSA-MP, and MSA-MI groups.

### 3.4. Comparison of Hippocampal Subfield Volumes among the HC, MSA-C, and MSA-P Groups

The left fimbria in the MSA-CP group was significantly higher than that in the HC group (64.6 ± 20.1 vs. 80.2 ± 28.8, *P*=0.012). Moreover, the hippocampal subfield volume showed no significant difference between the MSA-C and MSA-P groups.

### 3.5. Association between Neuropsychological Test Results and Hippocampal Subfield Volumes

The results of linear regression analysis showed that the right CA2/3 volume was significantly correlated with the FAB score (adjusted *R*^2^ = 0.282, *β* = 0.227, and *P*=0.041) and not correlated with the total/subscale ACE-III scores or WMS-R indices in patients with MSA. The left fimbria volume was not correlated with the FAB and total/subscale ACE-III scores or WMS-R indices.

## 4. Discussion

The current study included patients with MSA at the early disease stage, with an average disease duration of <2 years, and quantitatively compared hippocampal subfield volume changes in the MSA-CI, MSA-CP, and HC groups. Our findings showed that the right CA2/3 volume in MSA-CI was smaller than that in HCs and that the left fimbria volume in MSA-CP was larger than that in HCs. Moreover, the FAB score was associated with the right CA2/3 volume in patients with MSA. When MSA was divided into MSA-MI and MSA-MP based on WMS-R results, no significant differences were observed in hippocampal subfield volumes among groups.

Neurodegeneration in limbic structures including CA2/3 is associated with cognitive impairment of MSA. The current study showed a lower right CA2/3 volume in patients with MSA-CI than in HCs. Furthermore, the right CA2/3 volume significantly correlated with the FAB score. Previous clinicopathological studies have repeatedly demonstrated that the NCI burden in limbic regions contributes to the occurrence of cognitive impairment in MSA [5–10, 19]. Several studies with a semiquantitative detailed assessment of pathological findings in the medial temporal region have demonstrated severe NCI burden in the hippocampal subregions, including CA2/3 [7, 9, 10]. In another study using automated segmentation of hippocampal subfield techniques, volume reduction was detected in a larger number of hippocampal subfields, including CA2/3, in patients with MSA with mild cognitive impairment [13]. The wider range of detecting volume reduction of hippocampal subfields than in the present study may be due to the inclusion of an MSA cohort with an average disease duration of 4 years, which is longer than that of the MSA cohort in this study. Conversely, globular NCIs in the neocortex were associated with cognitive impairment [20]. Furthermore, especially at the early disease stages, previous imaging and morphological data have suggested that deafferentation from subcortical structures and cortical pathology may play a role in cognitive dysfunction [4]. An MRI examination using voxel-based morphometry and diffusion tensor imaging has shown that patients with MSA with cognitive impairment exhibited a significant widespread microstructural cerebral white matter involvement in contrast to reduced cerebral gray matter volume [21]. Recent studies that performed resting-state functional MRI to characterize cognition-related network alterations in patients with MSA have demonstrated that disruptions of the dorsolateral prefrontal cortex (DLPFC)-default mode network, DLPFC-insula network, and cerebello-cerebral networks were associated with cognitive impairment in patients with MSA [22–24]. The idea that these network disruptions are the substrate for cognitive dysfunction is consistent with a study that found no pathological differences in the cortical or limbic regions between patients with MSA with and without cognitive dysfunction [25]. Therefore, intrinsic limbic structure degeneration appears to be one of several substrates, rather than the only substrate, for cognitive dysfunction in MSA.

Memory impairment due to focal hippocampal degeneration may not be the predominant substrate of cognitive dysfunction in MSA. Although memory and visuospatial domains are sometimes impaired, executive functions and fluency are most commonly affected in patients with MSA [4]. In the current study, memory impairment was also observed in the group with cognitive impairment classified based on FAB results; however, no association was detected between memory scores and the hippocampal subfield volume, which decreased in the group with cognitive impairment. Moreover, no significant volume reduction in hippocampal subfields was observed in MSA-MI classified based on WMS-R results. Another hippocampal subfield study found an association between language, abstract function, visuospatial/executive function, and hippocampal subfield volume, which decreased in patients with MSA with mild cognitive impairment [13]. Previous pathological studies revealed that a subset of patients with MSA with cognitive impairment showed abundant NCIs in the frontotemporal lobes, including medial temporal regions with and without frontotemporal dementia-like clinical characteristics [5–7, 10]. These findings suggest that hippocampal degeneration in MSA may be accompanied by frontal cortical degeneration and does not appear to be solely associated with memory impairment. Conversely, a pathological study with a larger cohort of patients with MSA showed that the NCI burden in the hippocampus and parahippocampus was associated with the occurrence of memory impairment in MSA [9]. However, despite the presence of unavoidable limitation of a retrospective postmortem study, not all patients underwent formal and systematic cognitive assessment, and the interval between cognitive assessment and autopsy widely varied in this previous study. Moreover, two-thirds of patients with MSA with impaired memory in the previous study had frontal-subcortical dysfunction, such as executive dysfunction, impaired processing speed, personality change, disinhibition, and stereotypy, in addition to impaired memory. Interregional correlations for a load of neural inclusion pathology have been reported in the hippocampus and basal forebrain/hypothalamus [20], and the association between global cognitive scores and cerebello-amygdaloid/parahippocampal networks has been reported [22]. Therefore, although a small number of MSA patients may present with pure memory deficits due to localized hippocampal degeneration, the hippocampus may be associated with cognitive dysfunction in MSA through multihit degeneration in the hippocampus and other cortical regions, including the frontal lobe, and network abnormalities between the hippocampus and other regions, including the cerebellum.

The fimbria is in direct continuity with the fornix and is occasionally described as a fornix component [26]. In the present study, we hypothesized that increased fimbria volume in MSA-CP or MSA-C is a compensatory mechanism similar to that reported in the fornix. A study using diffusion tensor imaging showed local increases of fractional anisotropy in the fornix in nonpsychotic relatives compared to patients with schizophrenia and HCs, and this finding was postulated to be a compensatory mechanism to protect against psychosis among relatives [27]. Another study using diffusion tensor imaging in cognitively normal participants divided into amyloid positive, amyloid intermediate, and amyloid negative groups based on results of amyloid positron emission tomography showed higher fraction anisotropy in the fornix in amyloid positive participants than in amyloid negative participants [28]. This finding could be interpreted as a compensatory mechanism in Alzheimer's disease before a cognitive decline. However, no significant association between fimbria volume and cognitive function was found in the current study, and further studies with a large cohort of patients with MSA are needed to confirm our speculation.

Limitations of our study are as follows: First, patients with MSA were clinically diagnosed without postmortem confirmation; therefore, some of these patients may be misdiagnosed. Second, the same detailed cognitive assessment performed in patients with MSA was not performed in HCs. Finally, there is no multiple comparison correction for comparisons of all subfield numbers, so type 1 errors cannot be ruled out.

## 5. Conclusions

In this study, the hippocampal subfield volume is decreased in patients with MSA-CI even at the early disease stages. The hippocampal subfield region with decreased volume was detected in patients with MSA-CI and was associated with cognitive function assessed by FAB. These findings indicate that specific structural changes in the hippocampus might be associated with cognitive deficits in MSA.

## Figures and Tables

**Table 1:** Demographic data and neuropsychological results of all groups.

Group	MSA-CI	MSA-CP	HC	*P*value group comparison	*P* value MSA-CI vs. MSA-CP	*P* value MSA-CI vs. HC	*P* value MSA-CP vs. HC
Total no	40	35	68				
Sex (male/female)^a^	24/16	21/14	23/45	0.007	1.000	0.008	0.011
Age (years, median, range)^b^	70.8 (49–84)	57.2 (46–78)	66.4 (42–83)	<0.001	0.0226	0.0566	<0.001
Education (years, median, range)^b^	12.0 (9–22)	13.0 (12–16)	14.0 (9–18)	0.004	0.0789	0.0038	0.4209
eTIV (mean ± SD)^c^	1488487.5 ± 168621.4	1458655.3 ± 176432.5	1427808.7 ± 136626.6	0.148	NA	NA	NA
Disease subtype (MSA-C/MSA-P)^a^	25/15	19/16	NA	0.471	NA	NA	NA
Disease duration (years, median, range)^d^	1.6 (0.4–8.6)	1.9 (0.7–4.0)	NA	0.208	NA	NA	NA
UMSARS part 2 score (median, range)^d^	14.0 (7–28)	13.0 (4–28)	NA	0.062	NA	NA	NA
FAB (median, range)^d^	13.0 (5–14)	16.0 (15–18)	NA	<0.001	NA	NA	NA
ACE-III total score (median, range)^d^	82.0 (57–95)	92.0 (77–98)	NA	<0.001	NA	NA	NA
Attention/orientation (median, range)^d^	16.0 (11–18)	18.0 (15–18)	NA	<0.001	NA	NA	NA
Memory (median, range)^d^	20.5 (9–26)	23.0 (15–26)	NA	<0.001	NA	NA	NA
Fluency (median, range)^d^	8.0 (2–12)	11.0 (8–13)	NA	<0.001	NA	NA	NA
Language (median, range)^d^	25.0 (15–26)	26.0 (22–26)	NA	0.001	NA	NA	NA
Visuospatial (median, range)^d^	14.5 (6–16)	15.0 (13–16)	NA	0.004	NA	NA	NA
WMS-R indexes							
Attention and concentration (mean ± SD)^e^	89.5 ± 13.6	103.5 ± 12.6	NA	<0.001	NA	NA	NA
General memory (mean ± SD)^e^	84.5 ± 15.2	94.6 ± 12.8	NA	0.003	NA	NA	NA
Verbal memory (mean ± SD)^e^	84.4 ± 15.0	92.1 ± 12.7	NA	0.019	NA	NA	NA
Visual memory (median, range)^d^	91.5 (54–121)	106.0 (61–121)	NA	0.003	NA	NA	NA
Delayed recall (mean ± SD)^e^	83.5 ± 16.0	95.4 ± 14.9	NA	0.001	NA	NA	NA
SDS (mean ± SD)^e^	43.8 ± 7.3	42.5 ± 7.5	NA	0.473	NA	NA	NA

MSA-CI, cognitively impaired multiple system atrophy; MSA-CP, cognitively preserved multiple system atrophy; eTIV, estimated total intracranial volume; SD, standard deviation; NA, not applicable; UMSARS, Unified Multiple System Atrophy Rating Scale; FAB, Frontal Assessment Battery; ACE-III, Addenbrooke's Cognitive Examination III; WMS-R, Wechsler Memory Scale-Revised; SDS, Zung Self-Rating Depression Scale. ^a^Chi-squared test (post hoc chi-squared tests adjusted for multiple comparison: *P* < 0.05/3=0.0167). ^b^Nonparametric test (Kruskal–Wallis one-way ANOVA with the post hoc Steel–Dwass test). ^c^Parametric test (one-way ANOVA). ^d^Mann–Whitney *U* test. ^e^Student's *t*-test.

**Table 2:** Group comparison of hippocampal subfield volume.

	MSA-CI	MSA-CP	HC	*F* value	Partial eta^2^	*P* value		
	*n* = 40	*n* = 35	*n* = 68			MSA-CI vs. MSA-CP	MSA-CI vs. HC	MSA-CP vs. HC
Left hippocampal tail	521.2 ± 68.4	565.4 ± 61.4	531.8 ± 67.2	1.504	0.022	0.294	1.000	0.534
Left subiculum	426.0 ± 67.6	447.5 ± 45.0	433.4 ± 50.2	0.067	0.001	1.000	1.000	1.000
Left CA1	580.0 ± 84.6	626.6 ± 77.9	603.4 ± 80.2	2.285	0.033	0.139	0.254	1.000
Left hippocampal fissure	166.7 ± 30.2	167.7 ± 30.2	160.1 ± 34.5	1.278	0.018	0.357	1.000	0.753
Left presubiculum	320.7 ± 50.3	322.9 ± 41.9	321.0 ± 46.4	0.131	0.002	1.000	1.000	1.000
Left parasubiculum	65.4 ± 19.2	62.9 ± 15.8	63.8 ± 17.4	0.146	0.002	1.000	1.000	1.000
Left molecular layer	513.0 ± 73.3	549.1 ± 49.2	531.0 ± 61.1	1.278	0.018	0.491	0.467	1.000
Left GC-ML-DG	262.1 ± 39.3	278.6 ± 23.9	271.0 ± 35.2	1.641	0.024	0.659	0.226	1.000
Left CA2/3	192.6 ± 32.6	199.5 ± 23.4	196.3 ± 28.8	1.758	0.025	0.548	0.204	1.000
Left CA4	229.0 ± 33.6	238.4 ± 20.8	234.2 ± 29.2	1.209	0.017	1.000	0.373	1.000
Left fimbria	63.5 ± 30.7	83.0 ± 27.1	64.6 ± 20.1	3.139	0.044	0.965	0.553	0.046
Left HATA	52.0 ± 8.5	56.0 ± 7.9	53.5 ± 8.8	2.111	0.030	0.164	0.298	1.000
Left whole hippocampus	3225.6 ± 417.0	3429.9 ± 281.6	3304.1 ± 334.4	1.289	0.019	0.381	0.634	1.000
Right hippocampal tail	560.7 ± 86.8	598.2 ± 64.5	559.2 ± 71.4	0.742	0.011	0.865	1.000	0.824
Right subiculum	436.9 ± 60.0	462.9 ± 45.2	437.4 ± 53.4	1.146	0.017	0.651	1.000	0.471
Right CA1	634.0 ± 79.2	684.4 ± 84.4	644.2 ± 82.3	1.673	0.024	0.212	1.000	0.708
Right hippocampal fissure	194.9 ± 38.4	180.5 ± 35.4	169.5 ± 37.5	1.154	0.017	1.000	0.695	0.597
Right presubiculum	298.2 ± 47.9	312.0 ± 39.1	307.2 ± 41.0	0.588	0.009	1.000	0.849	1.000
Right parasubiculum	53.9 ± 16.7	55.1 ± 14.7	55.4 ± 13.3	0.730	0.011	1.000	0.754	1.000
Right molecular layer	537.3 ± 66.6	581.4 ± 54.4	552.8 ± 59.8	2.570	0.036	0.080	0.365	0.957
Right GC-ML-DG	273.7 ± 36.0	293.1 ± 29.6	282.6 ± 35.6	2.943	0.041	0.123	0.085	1.000
Right CA2/3	209.8 ± 32.5	218.5 ± 26.8	215.6 ± 30.1	3.673	0.051	0.125	0.029	1.000
Right CA4	239.4 ± 31.2	251.4 ± 26.0	244.1 ± 30.0	2.616	0.037	0.170	0.110	1.000
Right fimbria	58.0 ± 24.9	81.2 ± 30.2	62.9 ± 21.1	2.858	0.040	0.429	1.000	0.055
Right HATA	53.4 ± 8.7	58.5 ± 12.0	57.2 ± 10.1	1.776	0.025	0.713	0.187	1.000
Right whole hippocampus	3355.3 ± 397.8	3596.6 ± 305.4	3418.6 ± 356.7	2.236	0.032	0.112	0.509	0.929

MSA-CI, cognitively impaired multiple system atrophy; MSA-CP, cognitively preserved multiple system atrophy; HC, healthy control; CA, cornu ammonis; GC-ML-DG, granular cell-molecular layer-dentate gyrus; HATA, hippocampus-amygdala transition area.

## Data Availability

Any data not published within the article will be anonymously shared upon request from any qualified investigator.
